# Zolbetuximab combined with chemotherapy in a patient with CLDN18.2-positive advanced gastric cancer undergoing hemodialysis: a case report

**DOI:** 10.3389/fonc.2026.1798282

**Published:** 2026-05-12

**Authors:** Yuhan Zhao, Yuqi Jin, Linglin Fu, Difei Yao, Zitao Zhou, Ying Dong, Yinuo Tan

**Affiliations:** 1 Department of Medical Oncology, Key Laboratory of Cancer Prevention and Intervention, Ministry of Education, The Second Affiliated Hospital, Zhejiang University School of Medicine, Hangzhou, Zhejiang, China; 2 Zhejiang Provincial Clinical Research Center for Cancer, Hangzhou, China; 3 Cancer Center of Zhejiang University, Hangzhou, China; 4 Center for Medical Research and Innovation in Digestive System Tumors, Ministry of Education, Hangzhou, China; 5 Department of Infectious Diseases, The Second Affiliated Hospital, Zhejiang University School of Medicine, Hangzhou, China; 6 Department of Pharmacy, Second Affiliated Hospital of Zhejiang University School of Medicine, Hangzhou, China; 7 Research Center for Clinical Pharmacy, Zhejiang University, Hangzhou, China; 8 Department of Clinical Medicine, Hangzhou Normal University, Hangzhou, Zhejiang, China

**Keywords:** case report, CLDN18.2-positive gastric cancer, hemodialysis, irinotecan, zolbetuximab

## Abstract

**Background:**

Despite meaningful advances in systemic therapy, the prognosis of patients with unresectable advanced or metastatic gastric cancer remains poor. Targeted and immunotherapeutic approaches benefit only selected subpopulations, while the majority of patients continue to lack effective treatment options. Claudin 18.2 (CLDN18.2) has recently emerged as a promising therapeutic target, and the CLDN18.2-targeted monoclonal antibody zolbetuximab has demonstrated clinically meaningful efficacy in multiple international clinical trials. However, evidence for its use in patients with severe renal impairment requiring maintenance hemodialysis is lacking. In this population, conventional chemotherapy is often limited by altered pharmacokinetics and the risk of heightened toxicity, highlighting an important unmet clinical need.

**Case summary:**

We report the case of a 46-year-old woman with metastatic CLDN18.2-positive, HER2-negative gastric adenocarcinoma who subsequently developed end-stage renal disease (ESRD) secondary to tumor progression, necessitating regular hemodialysis. Following multidisciplinary evaluation, irinotecan combined with zolbetuximab was initiated on the basis of individualized adjustment of the hemodialysis schedule. The regimen was well tolerated, associated with stabilization of renal function, and resulted in a marked decline in serum CA19–9 accompanied by a significant shrinkage of peritoneal metastatic lesions on CT imaging, with sustained disease control during treatment.

**Conclusion:**

This case highlights that in patients with advanced gastric cancer undergoing hemodialysis, CLDN18.2-targeted therapy combined with chemotherapy may be feasible and safe with close laboratory monitoring and individualized dialysis management and may serve as a valuable reference for personalized treatment in gastric cancer complicated by renal failure.

## Introduction

Gastric cancer remains one of the most prevalent malignancies worldwide and continues to rank as the third leading cause of cancer-related mortality (1). Despite progressive advancements in systemic therapies, outcomes for patients with unresectable locally advanced or metastatic gastric or gastroesophageal junction (GEJ) adenocarcinoma remain suboptimal. While fluoropyrimidine- and platinum-based chemotherapy serves as the standard first-line backbone, its therapeutic benefit is modest, with median overall survival typically confined to less than one year. Although targeted therapies, such as trastuzumab for HER2-positive tumors, and immune checkpoint inhibition, such as nivolumab for selected PD-L1–expressing populations, have provided meaningful survival advantages, approximately 70% of gastric cancers are HER2-negative, and only a subset derive durable benefit from immunotherapy (2–5). Consequently, the identification of novel therapeutic targets capable of benefiting a broader patient population is a clinical priority.

Claudin 18.2 (CLDN18.2) is a tight junction protein physiologically restricted to gastric mucosal epithelial cells (6). During malignant transformation, structural disintegration and loss of epithelial polarity exposes CLDN18.2 on the tumor cell surface, rendering it a highly attractive and tumor-selective therapeutic target (7–9). Approximately 35%–40% of gastric/GEJ adenocarcinomas exhibit moderate-to-strong CLDN18.2 expression (10, 11). Zolbetuximab, a monoclonal antibody targeting CLDN18.2, has demonstrated clinically meaningful efficacy in phase III trials and has recently been approved in several countries as first-line treatment for patients with CLDN18.2-positive, HER2-negative advanced disease (12–15).

In real-world clinical practice, however, systemic treatment of patients with advanced gastric cancer complicated by severe renal impairment remains particularly challenging. Those requiring maintenance hemodialysis are frequently deemed ineligible for standard chemotherapy owing to concerns regarding nephrotoxicity, altered pharmacokinetics, and heightened risk of cumulative systemic toxicity. Most cytotoxic agents undergo renal elimination, and compromised renal function predisposes to drug accumulation and increased adverse events, considerably restricting therapeutic options. Clinical evidence supporting the safe implementation of targeted therapy in combination with chemotherapy in dialysis-dependent patients remains extremely scarce, and published experience specifically involving CLDN18.2-directed treatment is exceedingly rare.

Herein, we describe a patient with HER2-negative, strongly CLDN18.2-positive advanced gastric cancer who developed ESRD, ultimately requiring hemodialysis due to disease progression. Following multidisciplinary deliberation and individualized treatment planning, adjusted hemodialysis scheduling enabled delivery of irinotecan in combination with zolbetuximab, achieving favorable tolerability and sustained disease control.

## Case presentation

**Figure 1:**
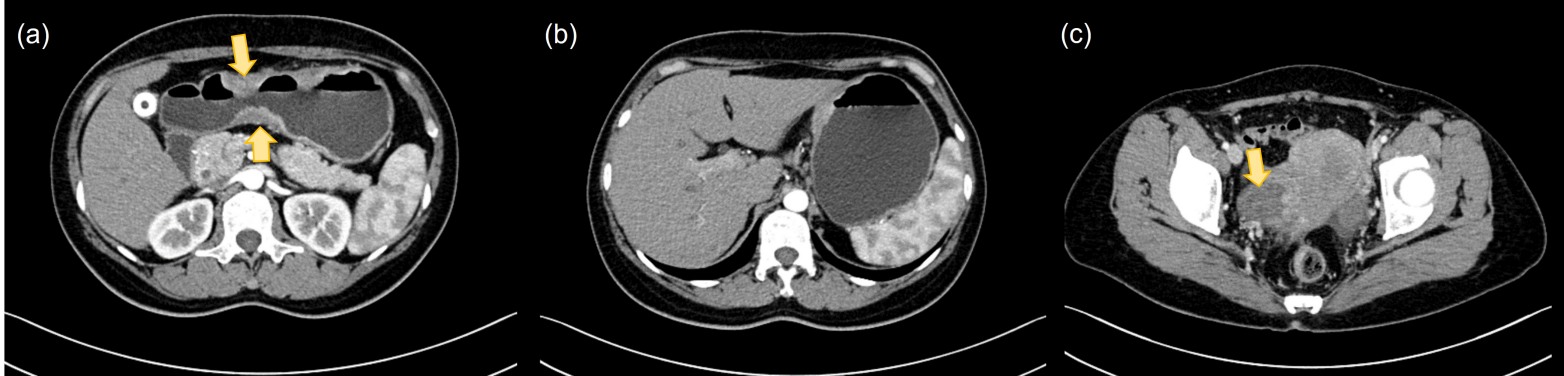
Contrast-enhanced abdominal computed tomography (CT) at initial diagnosis. **(a)** Imaging demonstrated advanced gastric cancer staged as T3N2, consistent with Borrmann type III (infiltrative ulcerative type). **(b)** Multiple morphologically abnormal small lymph nodes were observed along the lesser curvature of the stomach. **(c)** Bilateral ovarian enlargement was noted.

A 46-year-old woman presented in July 2022 with epigastric pain accompanied by nausea and vomiting. Contrast-enhanced abdominal computed tomography (CT) revealed an advanced gastric malignancy (clinical stage T3N2), characterized as a Borrmann type III infiltrative ulcerative lesion, with bilateral ovarian enlargement noted. She subsequently underwent palliative cytoreductive surgery, including total gastrectomy with lymphadenectomy and Roux-en-Y reconstruction, combined with bilateral adnexectomy, partial colectomy, and intraoperative hyperthermic intraperitoneal chemotherapy (HIPEC) on July 19, 2022. Histopathologic examination confirmed poorly to moderately differentiated adenocarcinoma of the gastric body (Lauren mixed type), with pathological staging of pT4bN3aM1 (Stage IV) and synchronous metastases involving both ovaries and the transverse colon (**Figure 1**). Immunohistochemistry demonstrated HER2 negativity (1+) and microsatellite stability (MSS), and the serum CA19–9 level at initial diagnosis measured 114.4 U/mL.

Postoperative adjuvant therapy was administered from August 2022 to April 2023. Treatment was initiated with CAPOX (capecitabine plus oxaliplatin) plus nivolumab for two cycles; however, owing to hepatotoxicity, the regimen was subsequently switched to FOLFOX (5-fluorouracil, leucovorin, and oxaliplatin) plus nivolumab for an additional seven cycles. Oxaliplatin was discontinued in February 2023 because of hypersensitivity, after which maintenance therapy with 5-fluorouracil and nivolumab was continued. During this period, serum CA19–9 levels normalized, and follow-up CT imaging revealed no obvious evidence of recurrent disease.

Progressive impairment of renal function was observed in June 2023. Imaging revealed bilateral ureteral wall thickening associated with hydronephrosis, findings that raised concern for immune-related ureteral inflammation. Concurrently, CA19–9 rose to 39.7 U/mL and serum creatinine increased to 147 μmol/L. Systemic antitumor therapy was therefore suspended, and bilateral ureteral stent placement was undertaken. Definitive disease progression was documented in August 2024, when serum CA19–9 increased to 384 U/mL and colonoscopic biopsy confirmed metastatic adenocarcinoma. Immunohistochemical evaluation demonstrated strong CLDN18.2 expression (80%), and cross-sectional imaging further revealed multifocal metastatic involvement of the peritoneum, rectum, and ileocecal region. Second-line therapy with nab-paclitaxel monotherapy was administered from September 2024 to January 2025 for seven cycles, during which CA19–9 levels initially normalized but subsequently rebounded. Throughout treatment, ureteral stents were regularly replaced, follow-up CT showed relatively stable mild bilateral pelvicalyceal dilatation, and renal function remained clinically stable with no significant fluctuation in serum creatinine.

**Figure 2:**
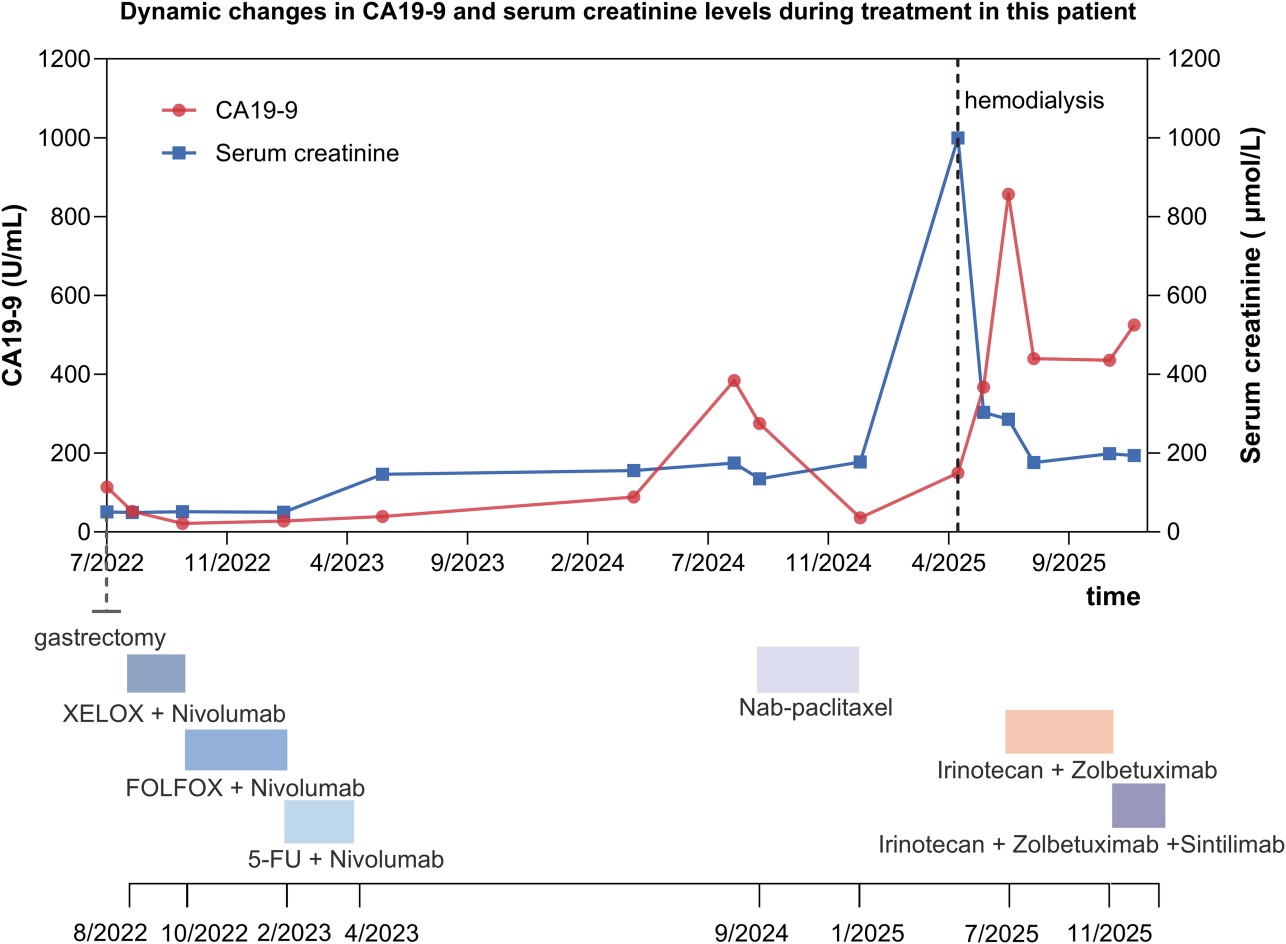
Dynamic changes in serum carbohydrate antigen 19-9 (CA19-9) and creatinine levels during systemic treatment.

A further decline in renal function ensued from March 2025 onward. Cystoscopic evaluation in April 2025 identified poorly differentiated adenocarcinoma involving the ureteral orifice, consistent with metastatic spread, and transurethral tumor resection with ureteral stent replacement was performed. By May 2025, serum creatinine had increased to approximately 1,000 μmol/L, necessitating commencement of maintenance hemodialysis. In June 2025, the patient developed subacute malignant small bowel obstruction. Exploratory surgery was initially intended for stoma diversion; however, diffuse miliary peritoneal metastases causing small intestinal contraction and stenosis were identified intraoperatively, necessitating partial small bowel resection with debulking of peritoneal lesions. Despite surgical intervention, serum CA19–9 continued to rise, reaching 856 U/mL. In the context of substantial tumor burden and radiographic evidence of multiple peritoneal metastatic nodules on CT, third-line systemic therapy with irinotecan combined with zolbetuximab was initiated on July 19, 2025. At treatment initiation, the patient weighed 37 kg with a height of 162 cm (body surface area, BSA 1.29 m²), on the basis of which irinotecan was administered at 100 mg on Day 1 of the first cycle and subsequently dose-reduced to 80 mg from the second cycle onward and thereafter maintained, whereas zolbetuximab was consistently delivered at 700 mg on Day 1 every three weeks. At the start of therapy, serum creatinine measured 360 μmol/L, and zolbetuximab was infused at a strictly controlled rate of approximately 40 drops per minute concomitantly with intravenous dexamethasone 5 mg, palonosetron, and fosaprepitant for antiemetic prophylaxis, during which the infusion was well tolerated without notable nausea or vomiting, and the hemodialysis schedule was carefully coordinated with systemic therapy. During the first treatment cycle, hemodialysis was performed one day prior to zolbetuximab infusion and again on Day 2 thereafter; from the second cycle onward, dialysis was adjusted to once a week and administered one day after chemotherapy. By the fifth treatment cycle, serum creatinine had stabilized at approximately 200 μmol/L, permitting discontinuation of dialysis. By January 8, 2026, the patient had completed nine cycles of irinotecan plus zolbetuximab. At reassessment in December 2025, serum CA19–9 was 525 U/mL, renal function remained stable, and follow-up CT scans revealed reduction of intra-abdominal lesions compared with baseline, consistent with a favorable radiographic response (**Figure 2**). The dynamic evolution of these imaging findings over time is presented in **Supplementary Material**.

## Discussion

This case illustrates that targeted therapy directed against CLDN18.2, when administered in combination with chemotherapy, may constitute a clinically feasible and potentially beneficial therapeutic strategy for patients with advanced gastric cancer requiring maintenance hemodialysis—an exceptionally challenging clinical population that is traditionally excluded from standard systemic therapy and lacks evidence-based treatment guidance. In the present patient, irinotecan plus zolbetuximab resulted in clinical benefit and survival prolongation despite dialysis dependence, suggesting that renal failure should not be an absolute contraindication to systemic anticancer treatment.

CLDN18.2, a member of the tight junction protein family, is physiologically expressed predominantly in normal gastric mucosal epithelial cells (6). In multiple gastrointestinal malignancies—including gastric, pancreatic, and biliary cancers—it becomes aberrantly overexpressed and exposed on tumor cell surfaces due to disruption of epithelial polarity, thereby providing an attractive and tumor-selective therapeutic target (16). Over recent years, CLDN18.2-directed therapy has made substantial advances. Zolbetuximab, the first approved CLDN18.2-targeted monoclonal antibody, has demonstrated robust clinical activity in several pivotal trials. The phase II FAST study first indicated that zolbetuximab combined with EOX chemotherapy (epirubicin, oxaliplatin, and capecitabine) could significantly prolong both progression-free survival and overall survival. These findings informed the design of subsequent phase III trials, which refined the biomarker threshold by increasing the required level of CLDN18.2 positivity from ≥40% to ≥75% of tumor cells displaying moderate-to-strong membranous staining, thereby enriching for patients most likely to benefit (9).

Two landmark phase III trials—the SPOTLIGHT and GLOW studies—subsequently evaluated zolbetuximab in combination with distinct chemotherapy backbones as first-line treatment for CLDN18.2-positive (≥75% moderate-to-strong membranous staining), HER2-negative unresectable locally advanced or metastatic gastric/GEJ adenocarcinoma. In SPOTLIGHT, zolbetuximab plus mFOLFOX6 significantly improved median progression-free survival (mPFS; 10.61 vs 8.67 months) and median overall survival (mOS; 18.2 vs 15.5 months), reducing the risk of death by 22%. Similarly, in GLOW, zolbetuximab plus CAPOX significantly prolonged mPFS (8.21 vs 6.80 months) and mOS (14.39 vs 12.16 months), corresponding to a 31% reduction in the risk of progression or death. Notably, although survival outcomes improved markedly, objective response rates were not substantially increased, suggesting that the predominant therapeutic effect of zolbetuximab may relate more to sustained disease stabilization than to rapid radiographic tumor shrinkage (14, 15). Updated data from the phase II ILUSTRO study presented at the 2026 ASCO Gastrointestinal Cancers Symposium further support CLDN18.2-directed combination strategies, showing that zolbetuximab plus nivolumab and mFOLFOX6 yields substantial activity as first-line therapy for CLDN18.2-positive gastric cancer. In the overall population, this triplet regimen achieved an objective response rate of 62.1% with an mPFS of 14.8 months, which increased to 18.0 months among patients with high CLDN18.2 expression (≥75%) and was further prolonged to 23.6 months in the subgroup characterized by both high CLDN18.2 expression (≥75%) and PD-L1 CPS ≥1. The safety profile remained manageable, thereby providing additional evidence supporting the first-line integration of CLDN18.2-targeted therapy with immune checkpoint inhibition and chemotherapy (17, 18). Looking ahead, therapeutic development is expected to increasingly emphasize rational combination strategies and individualized treatment approaches. Co-expression of CLDN18.2 and PD-L1 is relatively common in gastric cancer, offering a compelling biological rationale for combining CLDN18.2-targeted therapy with immune checkpoint inhibition. Multiple ongoing clinical trials are exploring this potential synergy. Moreover, next-generation CLDN18.2-directed modalities—including bispecific antibodies, antibody–drug conjugates, and CAR-T cell therapies—have already entered clinical development and hold promise for expanding therapeutic options and patient eligibility (19–22). A comprehensive overview of key clinical trials investigating zolbetuximab in CLDN18.2-positive solid tumors is summarized in **Table 1**.

**Table 1:** Key clinical trials of zolbetuximab in CLDN18.2-positive solid tumors.

Trial Name/NCT Number	Line	Phase	Patient Population	Enrollment	Interventions	Key results	Status	Reference
NCT01671774	≥2L	I	Adults with advanced CLDN18.2-positive (≥40% tumor cells) GC/GEJC	28	Arm 1: Zolbetuximab + ZA Arm 2: Zolbetuximab + ZA + low-dose IL-2 (1 mIU) Arm 3: Zolbetuximab + ZA + intermediate-dose IL-2 (3 mIU) Arm 4: Zolbetuximab	•Best confirmed response was SD (58%), no CR/PR; •mPFS: 37.3 weeks (zolbetuximab alone) •mOS: 60.9 weeks (zolbetuximab + ZA + intermediate-dose IL-2) •Most common zolbetuximab-related TRAEs: nausea (50%), vomiting (46.4%), fatigue (25%)	Completed	(37)
MONO (NCT01197885)	≥2L	II	Adults with advanced CLDN18.2-positive (≥50% tumor cells) GC/GEJC or EC	54	Zolbetuximab monotherapy	•ORR: 9% (4/43) In subgroup with CLDN18.2 ≥70%: ORR 14% (4/29) •Most common TRAEs: nausea (61%), vomiting (50%), fatigue (22%)	Completed	(38)
FAST (NCT01630083)	1L	RandomizedII	Adults with advanced CLDN18.2-positive (≥40% tumor cells) GC/GEJC or EC	252	Zolbetuximab + EOX vs EOX	•mPFS: 7.5 vs 5.3 months; HR = 0.44 (95% CI 0.29–0.67); P<0.0005 •mOS: 13.0 vs 8.3 months; HR = 0.55 (95% CI 0.39–0.77); P<0.0005 •ORR: 39% vs 25%; P = 0.034	Completed	(9)
GLOW (NCT03653507)	1L	Randomized III	Adults with CLDN18.2-positive (≥75% tumor cells), HER2-negative, locally advanced unresectable or metastatic GC/GEJC	507	Zolbetuximab + CAPOX vs Placebo + CAPOX	•mPFS: 8.21 vs 6.80 months; HR = 0.687 (95% CI 0.544-0.866); p=0.0007 •mOS: 14.39 vs 12.16 months; HR = 0.771 (95% CI 0.615-0.965); p=0.0118 •ORR: 42.5% vs 40.3% •Most common TEAEs: nausea (68.5% vs 50.2%), vomiting (66.1% vs 30.9%).	Active, not recruiting	(15)
SPOTLIGHT (NCT03504397)	1L	Randomized III	Adults with CLDN18.2-positive (≥75% tumor cells), HER2-negative, locally advanced unresectable or metastatic GC/GEJC	565	Zolbetuximab + mFOLFOX6 vs Placebo + mFOLFOX6	•mPFS: 10.61 vs 8.67 months; HR = 0.75 (95% CI 0.60-0.94); p=0.0066 •mOS: 18.23 vs 15.54 months; HR = 0.75 (95% CI 0.60-0.94); p=0.0053 •ORR: 48% vs 48% •Most common TRAEs: nausea (82% vs 61%), vomiting (67% vs 36%), decreased appetite (47% vs 33%)	Active, not recruiting	(14)
ILUSTRO (NCT03505320)	–	II	Adults with high or intermediate CLDN18.2-positive (≥50% tumor cells), HER2-negative, locally advanced unresectable or metastatic GC/GEJC	143	Cohort 1A (≥3L): Zolbetuximab monotherapy Cohort 2 (1L): Zolbetuximab + mFOLFOX6 Cohort 3A (≥3L): Zolbetuximab + pembrolizumab Cohort 4A + 4B (1L): Zolbetuximab + nivolumab + mFOLFOX6 Cohort 5: Zolbetuximab + FLOT	Cohort 4: •mPFS: overall: 14.8 months; CLDN18.2 high: 18.0 months; CLDN18.2 high + PD-L1 CPS≥1: 23.6 months; •mOS: CLDN18.2 high: NE; CLDN18.2 intermediate: 9.6months; •ORR: overall: 62.1%; CLDN18.2 high: 68.1%	Active, not recruiting	(17) (18)
LUCERNA (NCT06901531)	1L	Randomized III	Adults with CLDN18.2-positive (≥75% tumor cells), HER2-negative, PD-L1 CPS ≥1, locally advanced unresectable or metastatic GC/GEJC.	500 (Estimated)	Zolbetuximab + pembrolizumab + CAPOX/mFOLFOX6 vs Placebo + pembrolizumab + CAPOX/mFOLFOX6	–	Recruiting	(39)
RAINSPOT (NCT06962137)	2L	II	Adults with CLDN18.2-positive (≥75% tumor cells) metastatic gastroesophageal adenocarcinoma	100 (Estimated)	Zolbetuximab + paclitaxel + ramucirumab	–	Recruiting	–
NEO-CLAUD (NCT06732856)	Neoadjuvant	Ib/II	Adults with CLDN18.2-positive (≥75% tumor cells), newly diagnosed, potentially resectable GC/GEJC	57 (Estimated)	Zolbetuximab + DOS	–	Recruiting	
NCT06396091	1L	I	Adults with CLDN18.2-positive (≥75% tumor cells) metastatic PC	12 (Estimated)	Zolbetuximab + mFOLFIRINOX	–	Active, not recruiting	
NCT03816163	1L	RandomizedII	Adults with CLDN18.2-positive (≥75% tumor cells) metastatic PC	396	Zolbetuximab + GN vs GN	–	Active, not recruiting	(40)

GC, gastric adenocarcinoma; GEJC, gastroesophageal junction adenocarcinoma; EC, esophageal adenocarcinoma; PC, pancreatic adenocarcinoma; EOX, epirubicin, oxaliplatin, capecitabine; FLOT, fluorouracil, leucovorin, oxaliplatin, docetaxel; DOS, docetaxel, oxaliplatin, S-1; CAPOX, capecitabine, oxaliplatin; mFOLFOX6, modified fluorouracil, leucovorin, oxaliplatin; GN, gemcitabine + nab-paclitaxel; ZA, zoledronic acid; IL-2, interleukin-2; TRAEs, treatment-related adverse events; TEAEs, treatment-emergent adverse events; ORR, objective response rate; mPFS, median progression-free survival; mOS, median overall survival; SD, stable disease; NR, not reached; NE, not estimable.

Zolbetuximab, the first-in-class monoclonal antibody targeting CLDN18.2, is eliminated via linear and time-dependent pathways within a two-compartment model (23, 24). Gastrointestinal adverse events—particularly nausea and vomiting—constitute the most frequently observed toxicities associated with zolbetuximab and typically occur early during treatment. Current clinical practice supports the use of intensive prophylactic antiemetic regimens incorporating NK1 receptor antagonists, 5-HT3 receptor antagonists, dexamethasone, and olanzapine (25). Available data suggest that standard dosing can be safely applied in patients with mild to moderate renal impairment, as renal dysfunction appears to have minimal impact on drug exposure (23, 24). Nevertheless, pharmacokinetic and clinical data in patients with severe renal impairment or those dependent on hemodialysis remain extremely limited.

Irinotecan, a cornerstone agent in gastrointestinal cancers, is a prodrug whose antitumor efficacy relies on its active metabolite, SN-38, which inhibits topoisomerase I, inducing DNA damage and apoptosis (26, 27). Regarding elimination pathways, renal excretion serves as an important but non-predominant route for irinotecan and its metabolites. Research data indicate that the excretion of irinotecan and related substances via urine and feces accounts for approximately 32.1% and 63.7% of the administered dose, respectively, establishing fecal excretion as the predominant route (26). Urinary excretion of SN-38 and its glucuronide SN-38G is minimal, collectively accounting for only 1–3% of the administered dose (28). Small-scale studies suggest that in ESRD patients undergoing hemodialysis (creatinine clearance <20 ml/min), the terminal elimination rate of SN-38 may be reduced to roughly one-tenth of that observed in individuals with normal renal function, although plasma concentrations of irinotecan and SN-38G generally remain stable. Additionally, SN-38 itself can be partially cleared by dialysis, with clearance rates ranging from approximately 27% to 50% depending on the surface area of the dialysis membrane (29). Consequently, most dialysis patients, except those carrying UGT1A1 mutations that cause dual impairment in SN-38 clearance (30), can tolerate irinotecan therapy with or without dose adjustment (31–34).

End-stage renal disease has traditionally been regarded as a relative contraindication to systemic chemotherapy due to impaired renal elimination and the consequent risk of drug accumulation and heightened toxicity. Hemodialysis serves as the principal renal replacement modality in such patients, and systemic treatment in this setting requires meticulous consideration of both individualized dose adjustment and optimal timing relative to dialysis to avoid premature drug clearance while preventing excessive accumulation (35). Limited case reports have suggested that irinotecan may be cautiously administered after dialysis with appropriate dose modification; however, prior clinical experience with zolbetuximab in dialysis-dependent patients has been essentially absent (36).

In the present case, the patient had CLDN18.2-positive advanced gastric cancer complicated by severe renal dysfunction. In the context of substantial tumor burden and rapidly progressive disease, we implemented a reduced-dose irinotecan regimen (100 mg on Day 1) combined with zolbetuximab (700 mg on Day 1), together with dynamic adjustment of dialysis frequency and timing based on serial renal function monitoring. Following treatment initiation, renal function did not further deteriorate; rather, serum creatinine stabilized, and the patient was ultimately able to discontinue dialysis, supporting the safety and clinical feasibility of this individualized treatment approach in this highly complex scenario.

## Conclusion

To the best of our knowledge, this is the first report of zolbetuximab combined with irinotecan being safely and effectively administered in a patient with advanced gastric cancer undergoing maintenance hemodialysis. Through individualized dose adjustment and dialysis scheduling, this approach achieved favorable tolerability and meaningful disease control. Nevertheless, as a single-case observation, these findings require validation in larger cohorts before generalized recommendations can be established.

## Data Availability

The original contributions presented in the study are included in the article/**Supplementary Material**, further inquiries can be directed to the corresponding author/s.
