# Supplementary material for: Zolbetuximab combined with chemotherapy in a patient with CLDN18.2-positive advanced gastric cancer undergoing hemodialysis: a case report
Source: Front Oncol. 2026 May 12;16:1798282. doi: 10.3389/fonc.2026.1798282 (PMC13201143; doi:10.3389/fonc.2026.1798282)
Supplement: Supplementary file 1 [file Presentation1.pptx]

## Slide 1
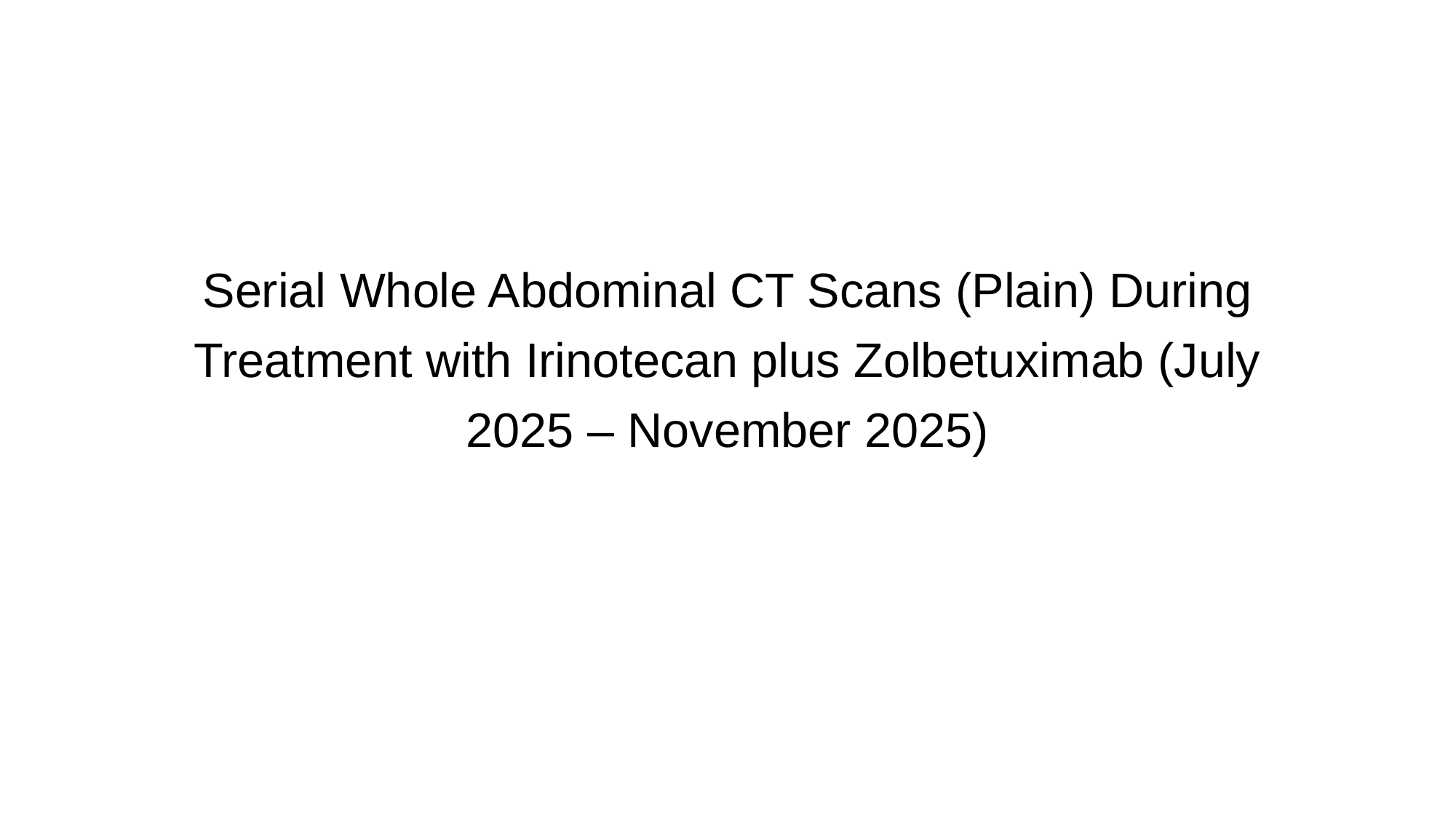

# Serial Whole Abdominal CT Scans (Plain) During Treatment with Irinotecan plus Zolbetuximab (July 2025 – November 2025)

## Slide 2
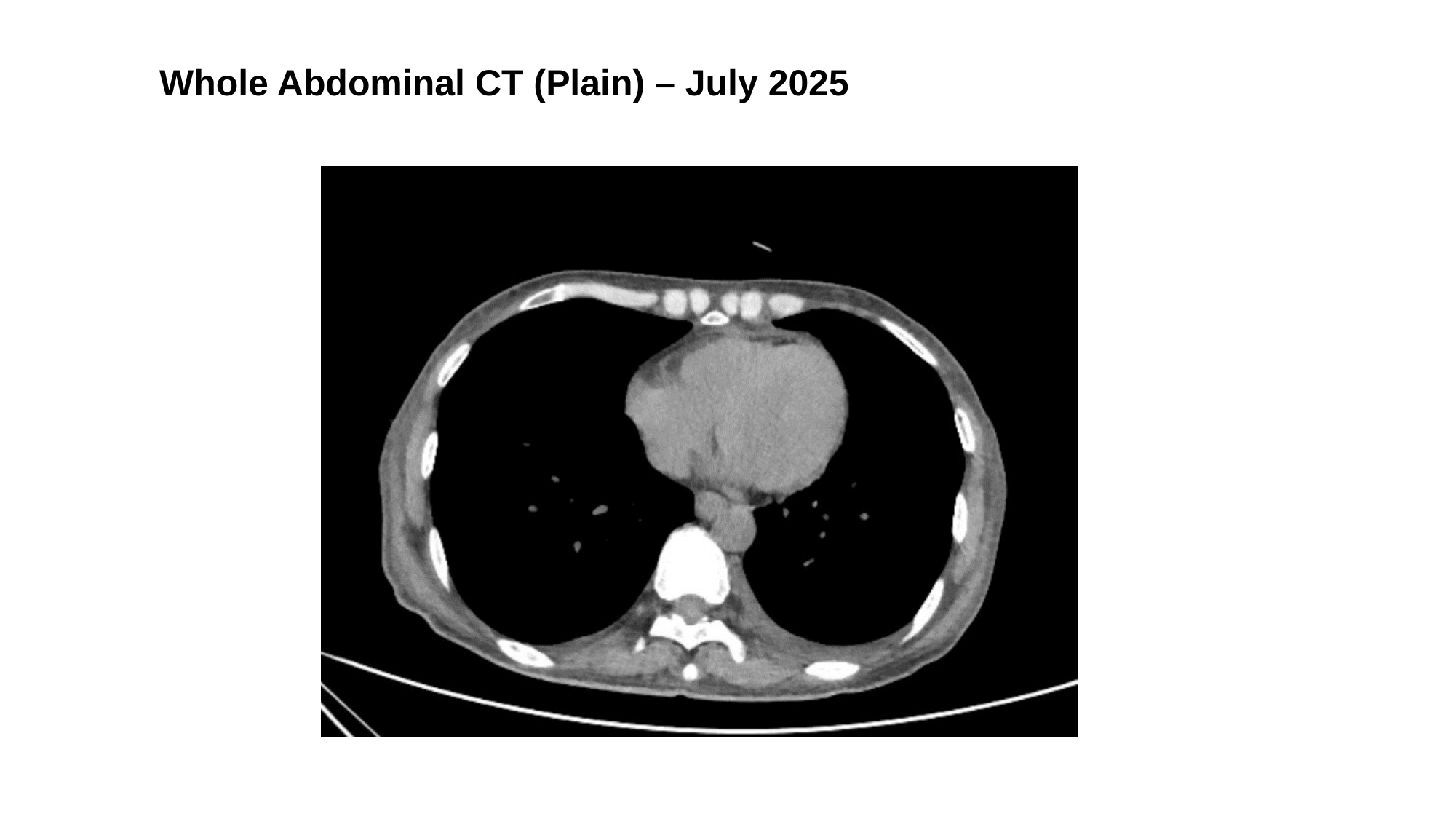

Whole Abdominal CT (Plain) – July 2025

## Slide 3
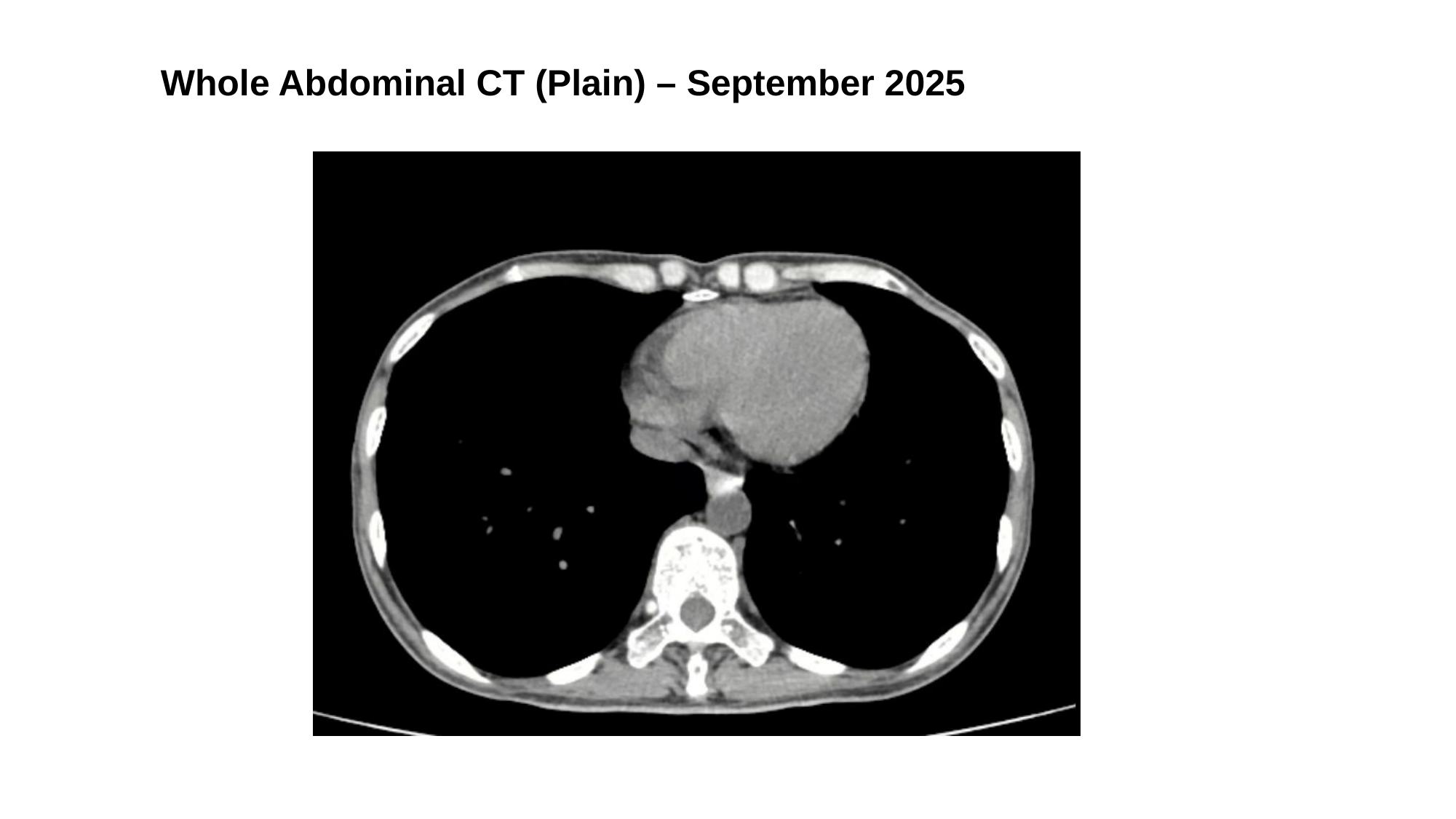

Whole Abdominal CT (Plain) – September 2025

## Slide 4
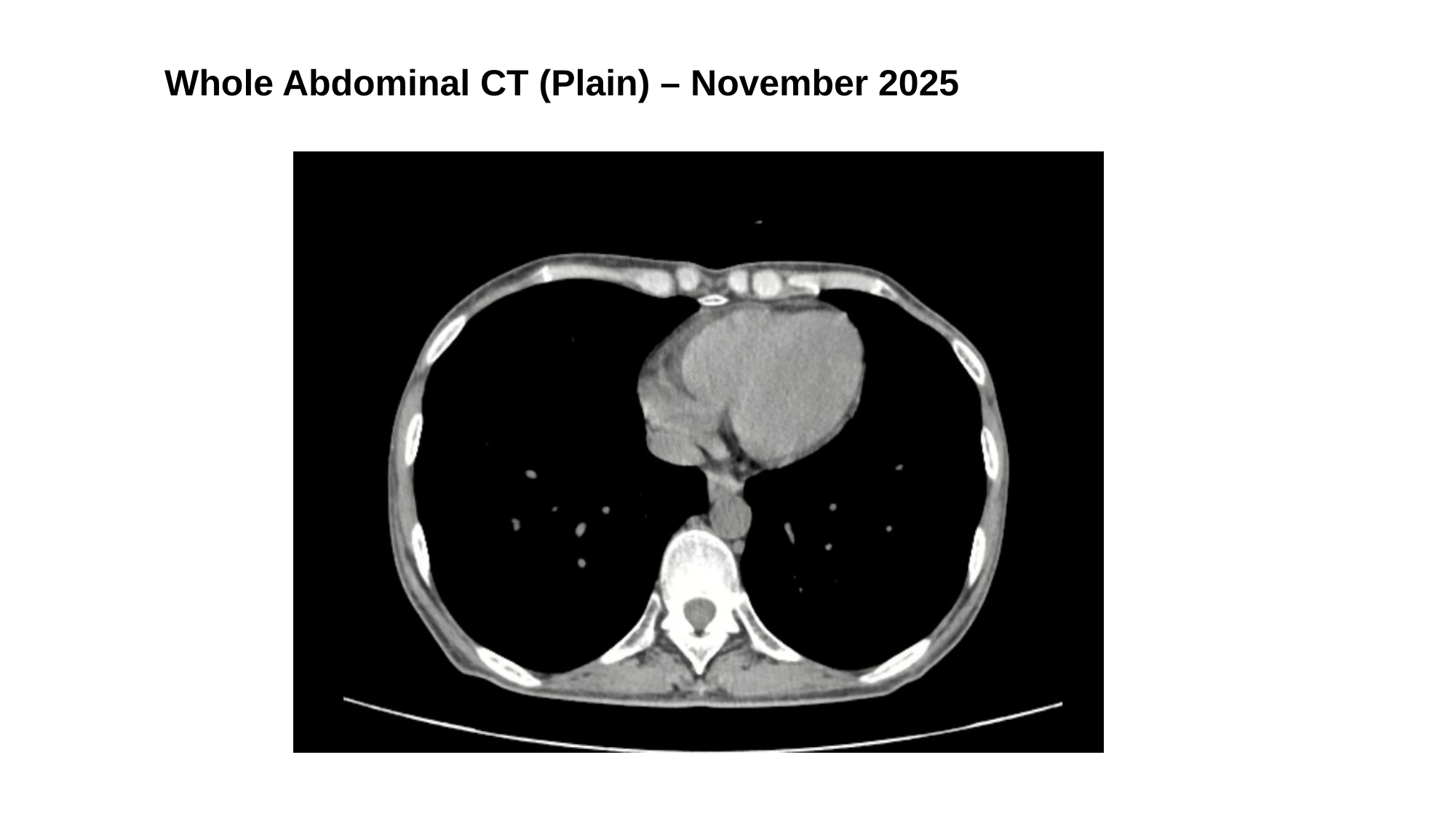

Whole Abdominal CT (Plain) – November 2025
